# A Glimpse of the Pathogenetic Mechanisms of Wnt/****β****-Catenin Signaling in Diabetic Nephropathy

**DOI:** 10.1155/2013/987064

**Published:** 2013-12-25

**Authors:** Li Xiao, Ming Wang, Shikun Yang, Fuyou Liu, Lin Sun

**Affiliations:** Department of Nephrology, 2nd Xiangya Hospital, Central South University, Changsha, Hunan 415800, China

## Abstract

The Wnt family of proteins belongs to a group of secreted lipid-modified glycoproteins with highly conserved cysteine residues. Prior results indicate that Wnt/**β**-catenin signaling plays a prominent role in cell differentiation, adhesion, survival, and apoptosis and is involved in organ development, tumorigenesis, and tissue fibrosis, among other functions. Accumulating evidence has suggested that Wnt/**β**-catenin exhibits a pivotal function in the progression of diabetic nephropathy (DN). In this review, we focused on discussing the dual role of Wnt/**β**-catenin in apoptosis and epithelial mesenchymal transition (EMT) formation of mesangial cells. Moreover, we also elucidated the effect of Wnt/**β**-catenin in podocyte dysfunction, tubular EMT formation, and renal fibrosis under DN conditions. In addition, the molecular mechanisms involved in this process are introduced. This information provides a novel molecular target of Wnt/**β**-catenin for the protection of kidney damage and in delay of the progression of DN.

## 1. Introduction

Diabetic nephropathy (DN) is a chronic kidney disease and is a major complication of diabetes mellitus. DN is also the main cause of end-stage renal disease worldwide [1]. It is known that the main pathological feature of DN includes mesangial expansion, podocyte loss, increased thickness of the basement membrane, and glomerular and tubular cell injury, which results in glomerulosclerosis and interstitial fibrosis [2, 3]. The pathogenesis of DN is very complicated and many different cells, molecules, and multifactors are involved in this process [4]. Metabolic factors, such as hyperglycemia and subsequent AGE production, oxidative stress, and the activation of several signaling pathways are thought to be the driving force and play a key role in the injury of renal cells and of extracellular matrix overproduction in DN [5–7].

Numerous studies have demonstrated that the TGF-*β*/Smad signaling pathway, a well-known profibrotic pathway in renal fibrosis, plays a key role in the progression of DN. In addition to the TGF-*β*/Smad pathway, many non-Smad pathways, such as PI3K/Akt, p38 MAPK, and JAK/STAT signaling, are also known to contribute to the development of DN [7]. Although these molecules and pathways have been identified, the pathogenesis of DN is not fully understood and there is no current satisfactory form of treatment. Thus, new signaling pathways have received much attention in DN studies. Encouragingly, accumulating studies have shown that Wnt/*β*-catenin signaling, a multifunctional pathway, is involved in renal cell injury, including mesangial cells, podocytes, and tubular cell damage, and this pathway has also been associated with tubular interstitial fibrosis in DN [8–10], which has attracted intense interest in the exploration and elucidation of the effect of Wnt/*β*-catenin signaling in DN progression.

### 1.1. Wnt/*β*-Catenin Signaling

**Figure 1:**
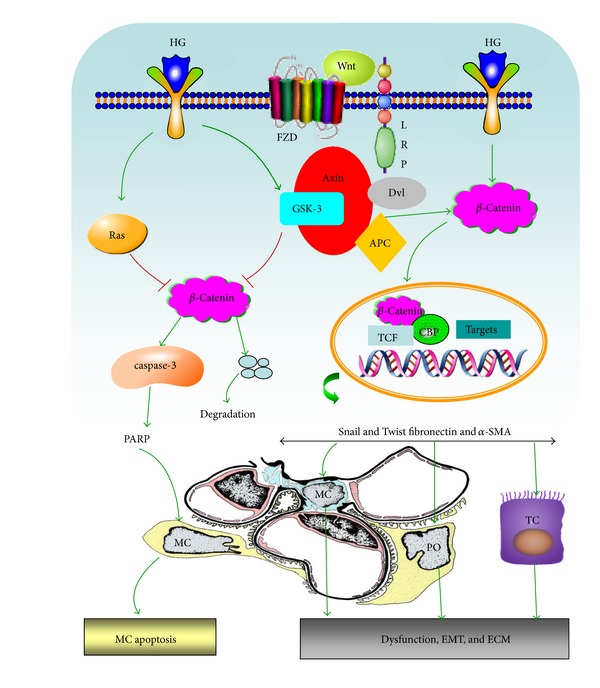
A schematic drawing depicting the role of wnt signaling in diabetic nephropathy. And High glucose induced Ras and GSK-3*β* activation, which leads to destabilization of *β*-catenin for degradation and subsequently promotes mesangial cells apoptosis through activation of caspase-3 and PARP. On the other hand, high glucose stimulates some Wnts secretion, increased intracellular Wnts level could activate its receptors FZD and LRP cause to the recruitment of the Dvl and Axin, inhibiting *β*-catenin phosphorylation and its degradation. Accumulated *β*-catenin then translocates to the nucleus and regulates the transcription activity of target genes such as Snail, a-SMA by TCF and CBP. In mesangial cell, the stabilized *β*-catenin induces fibronectin and *α*-SMA expression, which is suggested to be involved in epithelial-mesenchyml phenotypic transition (EMT) and contributes to kidney fibrosis under DN condition. In podocyte, it induced the Snail and suppressed nephrin expression, leading to podocyte EMT and dysfunction eventually leads to proteinuria and kidney fibrosis. In addition, high glucose activation of Wnt/*β*-catenin induces EMT formation and fibrosis in tubular cells by increased Snail and Twist expression. All of them eventually cause renal and tubular intestinal fibrosis in DN.

The Wnt family of proteins belongs to a group of secreted lipid-modified glycoproteins, which contain highly conserved cysteine residues in its amino acid sequence. There are 19 different Wnt proteins that have been identified in humans and mice [11]. It is known that secreted Wnt molecules can bind to cell surface receptors, such as frizzled and the low-density lipoprotein receptor-related protein (LRP). The best characterized Wnt signaling pathway is the canonical Wnt/*β*-catenin signaling pathway. Under inactive condition, *β*-catenin is bound to Axin and adenomatous polyposis coli (APC) and interacts with glycogen synthase kinase-3*β* (GSK-3*β*) for its phosphorylation at N-terminal residues and then leads to ubiquitin-mediated proteasomal degradation of *β*-catenin [11]. In contrast, when Wnt signaling was activated by various intercellular stimulator, Wnt ligands could activate FZD and LRP targeting APC and Axin, leading to dephosphorylation of GSK-3*β* and the recruitment of the cytosolic proteins Dishevelled (Dvl), which inhibit phosphorylation of *β*-catenin then cause to *β*-catenin accumulate. Subsequently, *β*-catenin translocates to the nucleus and activates T-cell factor (TCF) and lymphoid enhancer factor (LEF) to regulate the expression of Wnt target genes (Figure 1) [12]. There are several secreted protein antagonists of Wnt signaling that have been previously identified, including secreted Frizzled-related proteins (sFRPs) and Dickkopf (DKK) protein, which are thought to function as negative feedback regulators of the Wnt/*β*-catenin pathway [13, 14]. Numerous studies have demonstrated that Wnt ligands via their cell membrane bound receptors and coreceptors exert many fundamental physiological and pathophysiological functions in multiple organs and cell lineages, including organogenesis, tumorigenesis, and fibrosis, among others [15]. Importantly, it has been reported that the Wnt pathway can cross talk with transforming growth factor-*β* (TGF-*β*)/Smad, Notch pathways and connective tissue growth factor (CTGF). These interactions play an important role in embryonic proliferation, differentiation, cell adhesion, cell survival and apoptosis and is involved in organ development and diseases [16–23], including various kidney diseases, particularly diabetic nephropathy [10, 23–25].

Recently, emerging evidence has suggested that Wnt/*β*-catenin pathway is activated under diabetic conditions. Zhou et al. showed that the levels of *β*-catenin and WNT proteins are upregulated in the kidney tissues of both type 1 and type 2 diabetic animal models. However, insulin could attenuate the activation of WNT signaling via lowering blood glucose levels [10]. In addition, high glucose activated WNT signaling in cultured human renal proximal tubular epithelial cells, whereas inhibition of WNT signaling using the anti-LRP6 antibody ameliorated renal inflammation reduced proteinuria and ameliorated fibrosis [10]. Moreover, activation of the Wnt/*β*-catenin signaling pathway has been demonstrated to play a role in the pathogenesis and progression of DN, and multiple cells are thought to be involved in this process, including mesangial cells, podocytes, and tubular cells [8–10].

### 1.2. Wnt/*β*-Catenin and Mesangial Cell Injury

Accumulating studies have shown that mesangial cells (MCs) play an important role in the structure and function of glomerular maintenance, which supports capillary loops and regulates glomerular filtration [26]. Substantial evidence has shown that hyperglycemia or high glucose (HG) induces MC apoptosis, which has been attributable to the progression of DN [8, 25, 27, 28]. Furthermore, abnormal activity of the Wnt/*β*-catenin signaling pathway is involved in the regulation of morphological changes and pathogenesis in glomerular cells in DN, and its effect in HG-induced MCs apoptosis is particularly emphasized. Recently, Lin et al. found that HG inhibits the expression of Wnt4 and Wnt5a in cultured MCs, while increased GSK-3 expression and caspase-3 activities resulted in an increase in the level of MC apoptosis. In contrast, a reduction in HG-mediated caspase-3 cleavage and cell apoptosis was observed to inhibit GSK-3*β* activation or increase in nuclear *β*-catenin via transfection of Wnt4 or Wnt5a or stable *β*-catenin (S33Y) [8]. Similarly, simvastatin protected MCs from apoptosis when exposed to HG via inhibition of GSK-3*β* and restoration of Wnt4 and Wnt5a expression *in vitro* and *in vivo* [25]. Moreover, spironolactone significantly inhibited the apoptosis of rat MCs under hyperglycemic conditions *via* activation of the Wnt signaling pathway [26]. In addition, HG can induce Wnt5a/*β*-catenin destabilization and subsequently promotes caspase-3 and poly (ADP-ribose) polymerase cleavage, which subsequently caused apoptosis in cultured MCs [8]. These data indicated that sustaining Wnt/*β*-catenin signaling is beneficial in preventing HG-induced apoptosis of mesangial cells.

Recent studies have demonstrated cross talk between oxidative stress and Wnt/*β*-catenin signaling in MC cell survival induced by HG ambient. Lin et al. reported that HG can induce Ras and Rac1 activation, increase ROS production and destabilization of Wnt5a/*β*-catenin in MCs, and subsequently induce cellular apoptosis via the promotion of caspase-3 and poly (ADP-ribose) polymerase cleavage. However, this effect was abolished in cells transfected with either dominant-negative Ras (S17N) or dominant-negative Rac1 (T17N). Furthermore, stabilization of *β*-catenin via transfection of stable *β*-catenin (Delta45) and kinase-inactive GSK-3*β* has been shown to attenuate HG-mediated MCs apoptosis [28] indicating that HG increases apoptotic activity of mesangial cells, in which activated GSK-3*β* and inhibited Wnt5a/*β*-catenin signaling via the regulation of superoxide mediated by Ras and Rac1 activation was observed.

It is known that the transition of MCs plays a key role in the progression of DN. In addition to apoptotic regulation, accumulating studies have also demonstrated that Wnt/*β*-catenin is involved in the epithelial-mesenchymal phenotypic transition (EMT) of MCs under DN conditions [29]. TGF-*β*1 has been proposed as a prominent mediator of HG-induced EMT formation in various cells. In addition, *β*-catenin interacting protein 1 (CTNNBIP1) is a direct target of miR-215, which inhibits Wnt/*β*-catenin signaling. Mu et al. found that increased miR-215 expression suppressed CTNNBIP1 and activated Wnt/*β*-catenin, which subsequently promoted TGF-*β*-induced EMT formation of MCs in DN and is characterized by an increased expression of fibronectin and *α*-SMA. However, knockdown of miR-215 may reverse this phenotypic transition. Moreover, a previous study found that sustained Wnt signaling reduced c-Jun-dependent TGF-*β*1-mediated fibronectin accumulation in MCs [30], suggesting that miR-215 plays an important role in TGF-*β*1-mediated EMT formation of MCs via the CTNNBIP1/*β*-catenin pathway.

Recent evidence suggests that there is a complex relationship between CTGF/CCN family protein 2 (CTGF/CCN2) and the Wnt signaling pathway. Previous studies have demonstrated that CCN2 can modulate Wnt signaling by binding to LRP5/6. In addition, Wnt ligands increased CCN protein expression in *Xenopus Laevis* embryos [31]. Rooney et al. found that CTGF induced the phosphorylation of LRP6 and GSK-3*β*, which resulted in an accumulation of *β*-catenin and its nuclear localization, and activated the transcription factor TCF/LEF and increased MCs apoptosis via the regulation of the expression of Wnt targets. However, treatment with DKK-1, an endogenous LRP6 receptor antagonist or knockdown of LRP6 via siRNA, ameliorated CCN2-induced Wnt signaling activation in human MCs [32]. Moreover, both SERPINA3 K, a serine proteinase inhibitor, and DKK-1 blocked the overproduction of CTGF in cultured renal MCs exposed to HG [28]. Taken together, these data suggested a dual role of Wnt signaling in MCs apoptosis and EMT formation under DN conditions.

### 1.3. Wnt/*β*-Catenin and Podocyte Dysfunction

Emerging studies have demonstrated that Wnt/CTNNBIP1 plays an important role in the regulation and integration of cell adhesion, motility, and cell death and differentiation of glomerular podocytes. Proper *β*-catenin expression is essential to maintain the function of the glomerular filtration barrier [9]. Several studies have suggested that activated Wnt/*β*-catenin signaling promoted podocyte dysfunction in DN [9, 31, 33, 34]. In addition, podocyte injury is thought to induce glomerular albuminuria and subsequent glomerular injury in early onset DN [34]. Previous studies have also shown that the expression of WNT proteins, such as Wnt1, Wnt2B Wnt4, Wnt6, and Wnt16, is increased in the podocytes of a DN animal model [31]. Furthermore, in patients with DN, upregulation of Wnt1 and active *β*-catenin expression in podocytes were also observed [9]. Moreover, activation of Wnt/*β*-catenin resulted in podocyte dysfunction, whereas blockade of Wnt signaling by DKK1 ameliorated podocyte dysfunction and albuminuria [9], indicating that Wnt/*β*-catenin contributed to podocyte damage in DN pathogenesis. However, the underlying molecular mechanisms of this phenomenon remain poorly understood.

*In vitro* studies have shown that HG induced apoptosis and reduced the viability of differentiated mouse podocytes with an upregulation of TRPC6 and activation of the canonical Wnt signaling pathway, which was blocked when treated with DKK-1 [35]. These findings suggested that the Wnt/*β*-catenin pathway might potentially be activated during TRPC6-mediated podocyte injury in DN. Furthermore, angiotensin II (Ang II) plays an important role in promoting podocyte dysfunction and albuminuria. Encouragingly, there is some evidence demonstrating that Ang II induced Wnt1 expression and *β*-catenin nuclear translocation via calmodulin-dependent protein kinase II and cAMP response element-binding protein in cultured mouse podocytes. However, treatment with DKK-1 or *β*-catenin siRNA could partially block this effect [36]. In addition, Kato et al. reported that inhibition of CTNNB1 in cultured podocytes increased the expression of podocyte differentiation markers, which enhanced cell motility [31].

Podocyte-specific CTNNB1 knockout mice exhibited basement membrane damage, albuminuria, and an increased susceptibility to glomerular injury. Importantly, mice with either podocyte-specific deletion of CTNNB1 or podocyte-specific expression of DKK-1 showed an increased susceptibility to DN [31]. These findings suggested that *β*-catenin is a critical regulator of the maintenance of glomerular filtration barrier and its function.

Mechanisms of activated Wnt/*β*-catenin signaling-induced podocyte dysfunction in DN are complex and may involve an increase in the expression of either Wnt1 or stabilized *β*-catenin, which induced the transcription factor Snail and suppressed expression of the nephrin gene. These events subsequently resulted in podocyte dysfunction [9]. Emerging evidence has indicated that podocytes undergo EMT in DN, which is associated with proteinuria and kidney fibrosis of DN [9, 37]. During EMT, podocytes lose the expression of epithelial markers, including a decrease in the expression of occludens-1 (ZO-1), nephrin, and P-cadherin as well as zonula, which is accompanied by mesenchymal features, as reflected by the increased expression of desmin, fibroblast-specific protein-1, and matrix metalloproteinase-9 [37]. Interestingly, EMT also occurs in human biopsy samples of DN. Clinical evidence has indicated a loss in nephrin and ZO-1 expression in glomerular podocytes, whereas desmin, FSP1, MMP-9, and key EMT regulators, such as Snail and integrin-linked kinase (ILK), expression were significantly increased in DN patients [37–39], suggesting an active EMT formation in podocytes after DN. Recent studies have also shown that Ras-related C3 botulinum toxin substrate 1 (Rac1), combined with its major downstream effector p21-activated kinase 1 (PAK1), hastened EMT formation of podocytes via HG stimulation by promoting *β*-catenin and Snail transcriptional activities, which may be a potential mechanism in podocyte injury under diabetic conditions [40].

Furthermore, Li et al. indicated thatWnt/*β*-catenin pathway-mediated podocyte injury via the activation of transient receptor potential channel 6 (TRPC6) is essential for the proper regulation of podocyte structure and function, whereas inhibition of the Wnt/*β*-catenin pathway by DKK-1 downregulated HG-induced TRPC6 expression and ameliorated podocyte injury in DN [35]. In addition, administration of paricalcitol also ameliorated proteinuria in DN. Furthermore, the mechanisms underlying paricalcitol protection of podocyte damage from DN have demonstrated a physical interaction between the vitamin D receptor and *β*-catenin in podocytes, which inhibits *β*-catenin nuclear translocation and regulates target gene transcription [34].

### 1.4. Wnt/*β*-Catenin and EMT Formation in Renal Tubular Cells of DN

In addition to mesangial cells and podocytes, emerging studies have suggested that the renal tubule also exhibits a critical effect in DN development [41, 42]. Tubulointerstitial fibrosis (TIF) is an important effector underlying DN pathology and represents the final common pathway for DN with end-stage renal disease. It has been shown that EMT formation in tubular epithelial cells plays a key role in the progression for tubulointerstitial fibrosis in DN [43]. Although EMT occurs in tubular cells and attempts to evade apoptosis as a consequence of exposure to various pathophysiological stimuli, renal interstitial fibrosis and renal dysfunction eventually result, which has been clearly observed in the proximal tubule cells (PTC) of DN *in vitro* and *in vivo* [33, 44]. Recently, growing evidence has demonstrated that Wnt/*β*-catenin also contributes to the EMT formation of tubular cells in DN.

Zhou et al. showed that HG increased the expression of WNT proteins and induced Wnt/*β*-catenin activation in tubular epithelial cells [10]. Liu et al. also demonstrated that HG could cause the activation of Wnt/*β*-catenin and induce *β*-catenin target gene expression, including Snail and Twist, which suppressed E-cadherin expression and increased fibronectin, *α*-SMA and vimentin expression in tubular cells [33]. However, an inhibitor of Snail or *β*-catenin reversed HG-induced EMT of tubular cells [44], suggesting that Wnt/*β*-catenin mediated HG-induced EMT formation in tubular cells.

Interestingly, it has been reported there is cross talk between Wnt/*β*-catenin signaling and the TGF-*β*/Smad or non-Smad signaling pathway in TGF-*β* induced EMT formation in DN [33]. Hills et al. found that HG increased the secretion of TGF-*β*1 from cultured tubular cells and altered the expression of adhesion and adherens junction proteins, associated with decreased E-cadherin and connexin-43 production. In addition, HG also caused an impairment in cell adhesion and also decreased cell-to-cell communication [45]. Tian et al. demonstrated that *β*-catenin is required as a cofactor of Smad in TGF-*β*1-induced EMT of renal epithelial cells [46]. Further evidence obtained from Huang et al. showed that the Wnt/*β*-catenin signaling pathway participated in HG-induced renal tubular epithelial cell EMT formation, although this effect was inhibited by treatment with tanshinone IIA, which downregulated the activities of Wnt/*β*-catenin signaling [47]. Lee et al. found that transfection with Snail1 or *β*-catenin siRNA partially blocked HG-induced EMT relative protein expression in cultured primary proximal tubular cells. They also demonstrated that HG decreased PPAR*γ* activation, while the PPAR*γ* agonist troglitazone reversed HG-stimulated expression of GSK-3*β*, *β*-catenin and Snail1, and EMT relative proteins [44].

### 1.5. Wnt/*β*-Catenin and Renal Fibrosis

Surendran et al. found that Wnt/*β*-catenin activation in unilateral ureteral obstruction (UUO) mice was associated with matrix metalloproteinases-7, fibronectin, Twist, and c-Myc. However, treatment using recombinant sFRP4, an inhibitor of Wnt signaling, resulted in a decreased expression of these extracellular matrix (ECM) related genes and alleviated the progression of renal fibrosis [48]. In addition, Wnt4 mRNA and protein expression significantly increased in the proximal tubules of an animal model with acute kidney injury and activation of Wnt/*β*-catenin was involved in the cyst formation of polycystic kidney disease [49]. These data indicated that activated Wnt/*β*-catenin might be involved in various kidney injuries and renal fibrosis. Recent studies have also suggested that Wnt/*β*-catenin plays a pivotal role in renal fibrosis in DN.

Accumulation of ECM components in the mesangial area is a prominent hallmark of DN, which ultimately results in renal fibrosis [50, 51]. Several studies have shown that the Wnt/*β*-catenin pathway participated in TGF-*β*/Smad signaling-modulated ECM protein accumulation in the DN state [30, 52]. Ho et al. reported a downregulated expression of Wnt/*β*-catenin, which was accompanied with an increased expression of TGF-*β* and fibronectin in MCs exposed to HG, while this effect was alleviated in cells transfected with Wnt4, Wnt5a, and stable *β*-catenin [30]. In addition, Lin et al. also revealed that the expression of DKK1 were strongly up-regulated and was associated with increased TGF-*β* and fibronectin expression in both the renal tissue of STZ-induced diabetes rats and in cultured mesangial cells exposed to HG. However, knockdown of DKK1 attenuated mesangial matrix accumulation via the restoration of the activation of Wnt/*β*-catenin signaling under hyperglycemia conditions [52].

In addition, Zhou et al. found that the expression of Wnt and cytoplasmic *β*-catenin were up-regulated in proximal tubular epithelial cells under DN conditions *in vitro* and *in vivo*, which was accompanied by an increased expression of CTGF and fibronectin. Injection of LRP5 and LRP6 antibodies suppressed activation of the WNT pathway and decreased ECM formation in DN animal models, suggesting that WNT/*β*-catenin signaling might be involved in tubular-interstitial fibrosis in DN [10]. Surendran et al. also reported the potential role of Wnt4 in tubulointerstitial fibrosis. They found that Wnt4 was initially limited to the collecting ducts, with an induction of HG, and subsequently, Wnt4 was activated and emerged in the interstitial area [22]. In addition, treatment with paricalcitol inhibited *β*-catenin signaling via competition of TCF-4 and activated vitamin D receptor, then attenuated renal interstitial fibrosis [34]. In addition, Akhmetshina et al. showed that TGF-*β* was sufficient to activate the Wnt/*β*-catenin via p38-dependent mechanism and decreased the expression of DKK-1. A selective TBRI inhibitor could reverse the effect of TGF-*β* on Wnt/*β*-catenin and potentially reduce renal fibrosis [53].

## 2. Conclusions

The Wnt/*β*-catenin signaling pathway has been shown to be activated under DN conditions, which was involved in the apoptosis and EMT formation of mesangial cells, podocyte dysfunction, and tubular cells EMT, subsequently resulting in renal fibrosis and interstitial fibrosis. It has been suggested that sustained Wnt/*β*-catenin expression is essential for its protective role against cellular damage, while abnormal activation of Wnt/*β*-catenin results in adverse effects and promotes the progression of DN, suggesting that Wnt/*β*-catenin plays a dual role in cellular damage under DN conditions. The mechanism underlying the regulation of Wnt/*β*-catenin and its crosstalk with other signaling pathways involved in DN is very complex and requires further studies. On the basis of these data, targets of the Wnt/*β*-catenin signaling pathway may provide a new therapeutic method to prevent DN progression.
